# Measurements of Activity Coefficients at Infinite Dilution for Organic Solutes in the Ionic Liquids *N*-Ethyl- and *N*-Octyl-*N*-methylmorpholinium Bis(trifluoromethanesulfonyl)imide. A Useful Tool for Solvent Selection

**DOI:** 10.3390/molecules25030634

**Published:** 2020-02-01

**Authors:** Łukasz Marcinkowski, Joachim Eichenlaub, Elham Ghasemi, Żaneta Polkowska, Adam Kloskowski

**Affiliations:** 1 Department of Physical Chemistry, Faculty of Chemistry, Gdansk University of Technology, Narutowicza Str.11/12, 80-233 Gdansk, Poland; joachim.eichenlaub@gmail.com (J.E.); adaklosk@pg.edu.pl (A.K.); 2 Department of Analytical Chemistry, Faculty of Chemistry, Gdansk University of Technology, Narutowicza Str.11/12, 80-233 Gdansk, Poland; el.ghasemi74@gmail.com (E.G.); zanpolko@pg.edu.pl (Ż.P.)

**Keywords:** ionic liquids, morpholinium ILs, activity coefficient at infinite dilution, inverse gas chromatography, separation techniques

## Abstract

In recent years, many papers describing ionic liquids (IL) as promising solvents in separation techniques have been published. The conscious choice of appropriate ionic liquid as absorption media in effective extraction of selected types of analytes requires deeper understanding of the analyte−IL interactions. Therefore, intensive research is conducted to determine the values of activity coefficient at infinite dilution, which allows us to characterize the nature of these interactions. Based on the inverse gas chromatography retention data, activity coefficients at infinite dilution $\gamma_{13}^{\infty }$ of 48 different organic compounds in the ionic liquids *N*-ethyl-*N*-methylmorpholinium bis(trifluoromethanesulfonyl)imide [C_2_C_1_Mor][TFSI] and *N*-octyl-*N*-methylmorpholinium bis(trifluoromethanesulfonyl)imide [C_8_C_1_Mor][TFSI] were determined. The measurements covered a broad range of volatile organic compounds, including *n*-alkanes, *n*-alkenes, *n*-alkynes, alcohols, aldehydes, ketones, aromatic compounds and common polar solvents, representing different types of interactions. Activity coefficients at infinite dilution were measured in the temperature range from 313.15 to 363.15 K. The excess partial molar enthalpies and entropies at infinite dilution were determined. Selectivity at infinite dilution was also calculated for exemplary separation processes in the hexane/benzene system. The obtained results were analyzed and compared with literature data for ionic liquids containing the same anion [TFSI]¯ and different cations. The study results indicate that some potential applications of the investigated ionic liquids in separation problems exist.

## 1. Introduction

The fifth principle of Green Chemistry introduces a requirement for searching new substances that will become alternatives for toxic organic solvents, contributing in this way to the reduction of the unfavorable environmental impact of chemical processes [1]. For over three decades, chemists have been focused on the investigation of substances and processes that could be, at the same time, environmentally friendly and improve the efficiency of chemical methodologies. One such group, which nowadays can pretend to be called solvents of the future, is ionic liquids. Their unique properties fulfill almost all the principles of Green Chemistry. Ionic liquid synthesis is simple and can be reduced to minimum steps, as it is possible. Their chemical and physical properties can be tuned by right cation and anion. Their negligible vapor pressure and high thermal stability allow for their recovery and reuse. Pretending to be called green solvents, required continuation of studies on these promising compounds and successive deepening of knowledge about the Ionic liquids (ILs) are organic salts consisting of organic cation and organic/inorganic anion. A distinguishing feature of this type of compounds is a low melting point, which usually is less than 100 °C [2]. Due to the huge number of possible combinations of different cations and anions (10^18^), it is possible to model its properties, such as density, viscosity, refractive index and sound velocity, in dependence on the requirements of a given solution [3]. Their popularity in potential usage is a result of a unique set of properties, i.e., negligible vapor pressure and relatively high thermal stability. Ionic liquids found a place as solvents in sample preparation techniques [4,5,6] as stationary phases in chromatography [7,8] or electrolytes in electrochemistry [9,10]. 

Considering the use of an ionic liquid as a solvent, the solute-solvent interactions become the key issue. One such parameter is the value of the activity coefficient at infinite dilution: $\gamma_{13}^{\infty }$. In the last years, intensive research has been conducted to determine the values of $\gamma_{13}^{\infty }$, which are a perfect source of information in the characterization of volatile organic compound‒IL interactions [11,12,13,14]. The analysis of the obtained results may allow for direct choice of the best ionic liquid or may serve as a basis for creating theoretical models in order to predict crucial parameters, e.g., partial molar excess thermodynamic functions [15,16]. One of the most frequently employed techniques for determining is inverse gas chromatography (IGC). The methodology based on determining the retention times for equilibria systems where vapors of solutes are carried out by inert gas through a column filled with ionic liquid-coated solid support is commonly recognized as a reliable source of values [17,18,19]. 

In the present study, we investigated the interactions between the different types of organic compound groups and two new ionic liquids based on morpholinium cation [C_n_C_m_Mor]^+^ and bis(trifluoromethanesulfonyl)imide anion [TFSI]¯. The ionic liquids differed in the length of one alkyl chain attached to the nitrogen atom in cation; one of the ILs had CH_3_CH_2_− and CH_3_− groups [C_2_C_1_Mor]^+^, while the other one had CH_3_(CH_2_)_7_− and CH_3_− substituent [C_8_C_1_Mor]^+^. The cation based on the morpholine ring is a rather poorly described group [20,21,22]. Due to the presence of an oxygen atom in the structure of morpholine, cation is considered to be less toxic in comparison to imidazolium cations [23,24]. In the present study 48 solutes representing different groups of organic compounds (*n*-alkanes, *n*-alkenes, *n*-alkynes, alcohols, aldehydes, ketones and aromatic compounds) were used. Activity coefficients at infinite dilution were determined in the temperature range 313.15–363.15 K. Based on the relationship between $\gamma_{13}^{\infty }$ and temperature, the values of partial molar excess enthalpy at infinite dilution were estimated. Research conducted on both ionic liquids allowed us to analyze the effect of the chain length of alkyl substituent on the solute-solvent interactions and to determine its influence on the separation processes in aliphatic/aromatic hydrocarbon mixtures. 

## 2. Theory

The values of activity coefficients at infinite dilution $\gamma_{13}^{\infty }$ were calculated from the equation proposed by Everett [25] and Cruickshank et al [26]:

(1) $$\ln\gamma_{13}^{\infty }=\ln\left(\frac{n_{3}RT}{V_{N}P_{1}}\right)-\frac{P_{1}\left(B_{11}-V_{1}\right)}{RT}+\frac{P_{0}J_{2}^{3}\left({2B}_{12}-V_{1}^{\infty }\right)}{RT}$$

where indices 1, 2 and 3 denote a solute, carrier gas and ionic liquid, respectively; *T* is the column temperature (K), *R* is the gas constant (8.31446 J·K^−1^·mol^−1^), $P_{1}$ is the saturated vapor pressure of solute at temperature *T* (kPa), $P_{0}$ is the outlet pressure (kPa), *n*_3_ is the number of moles of ionic liquid at stationary phase (mol), *V_N_* is net retention volume of the solute (cm^3^), *B*_11_ is the second virial coefficient of pure solute (cm^3^·mol^−1^), *B*_12_ is the cross second virial coefficient (cm^3^·mol^−1^), $V_{1}$ is the molar volume of pure solute (cm^3^ ·mol^−1^), $V_{1}^{\infty }$ is the partial molar volume of solute at infinite dilution in the solvent (cm^3^ ·mol^−1^) and $J_{2}^{3}$ is the pressure correction term. The calculation was made under the assumption that $V_{1}$ = $V_{1}^{\infty }$.

The pressure correction term was calculated from the following equation:

(2) $$J_{3}^{2}=\frac{3}{2}\frac{{\left(P_{in}/P_{out}\right)}^{2}-1}{{\left(P_{in}/P_{out}\right)}^{3}-1}$$

where *P_in_* and *P_out_* denote the inlet and outlet pressure of the column (kPa). Net retention volume *V_N_* was calculated by applying a correction term resulting from the use of a bubble flow meter:

(3) $$V_{N}=J_{3}^{2}F(t_{r}-t_{0})\frac{T}{T_{m}}\left[1-\frac{P_{w}}{P_{out}}\right]$$

where *F* denotes the flow rate measured by means of bubble flow meter (cm^3^·min^−1^), *t_r_* is the solute retention time (min), *t*_0_ is the time of fly of gas not retained (min), *T_m_* is the temperature of bubble flow meter (ambient temperature) (K) and $P_{w}$ is the vapor pressure of water at ambient temperature (kPa).

The values of *B*_11_ and *B*_12_ were calculated with the Tsonopoluos method [27]. The values of $P_{1}$ were calculated by using the Antoine equation. The critical values, used in calculation, were obtained from the Knovel database [28]. The collected data and the values of *B*_11_*, B*_12_ and $P_{1}$ can be found in supplementary data (Tables S1 and S2 in the SM file).

The obtained values of $\gamma_{13}^{\infty }$ were correlated with temperature using the Gibbs-Helmholtz equation:

(4) $$\frac{\partial \ln\gamma_{13}^{\infty }}{\partial \left(1/T\right)}=\frac{\Delta H_{1}^{E,\infty }}{R}$$

Based on Equation (4), the values of excess partial molar enthalpy of mixing $\Delta H_{1}^{E,\infty }$ were computed under the assumption that $\Delta H_{1}^{E,\infty }$ does not change with temperature. 

The selectivity at infinite dilution *S_ij_* was calculated according to the following equation:

(5) $$S_{ij}=\frac{\gamma_{i3}^{\infty }}{\gamma_{j3}^{\infty }}$$

## 3. Results and Discussion

The values of activity coefficients at infinite dilution obtained in the specified temperature range and the associated uncertainties of measurement for [C_2_C_1_Mor][TFSI] and [C_8_C_1_Mor][TFSI] are presented in Table 1 and Table 2, respectively. The highest values of $\gamma_{13}^{\infty }$ were obtained for *n*-alkanes, which is typical for ionic liquids with a weak solute-solvent interaction.

**Figure 1:**
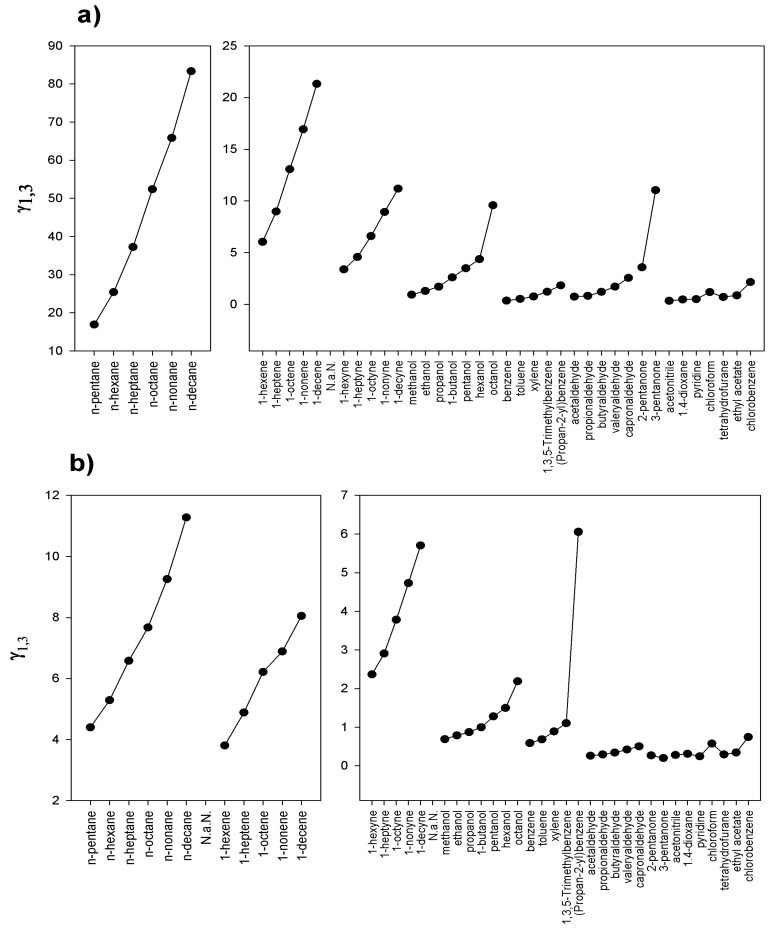
Comparison of $\gamma_{13}^{\infty }$ values at T = 328.15 K for different groups of solutes in (**a**) [C_2_C_1_Mor][TFSI] and (**b**) [C_8_C_1_Mor][TFSI].

Figure 1a,b present the comparison of $\gamma_{13}^{\infty }$ at T = 328.15 K for the studied ILs. The presence of double and triple bonds in a solute structure (presence of π electrons) significantly lowers the value of $\gamma_{13}^{\infty }$ for [C_2_C_1_Mor][TFSI]. This confirms the occurrence of stronger attractive interactions with the ionic liquid; the effect is more noticeable in the case of cation with ethyl substituent [C_2_C_1_Mor]^+^. Based on the obtained results, it can also be stated that the elongation of the alkyl chain decreases the interactions with the ionic liquid and, therefore, leads to the increased values of $\gamma_{13}^{\infty }$ for [C_2_C_1_Mor]-IL.

**Figure 2:**
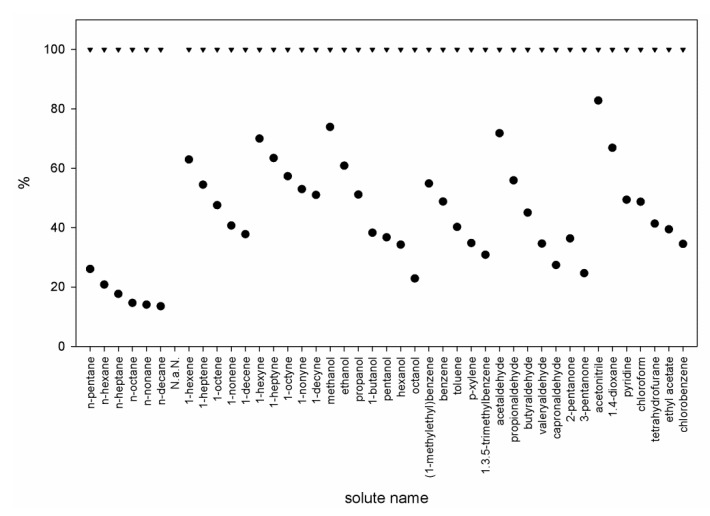
Relative comparison of $\gamma_{13}^{\infty }$ values for different solutes in (▲) [C_2_C_1_Mor][TFSI] and (●) [C_8_C_1_Mor][TFSI] (T = 328.15 K).

In general, the interactions of both ionic liquids with solutes decrease in the following sequence: *n*-alkynes; *n*-alkenes; *n*-alkanes. The analyses of $\gamma_{13}^{\infty }$ values determined for hexane and cyclohexane indicates that the role of molecular geometry of the researched compounds is significant. The lower value of $\gamma_{13}^{\infty }$ for cyclohexane in comparison to hexane corresponds to the difference in molar volumes of these compounds, and, at the same time, its more compact structure favors interactions with solvent. The influence of number and type of substituted groups can also be observed in the case of (1-methylethyl)benzene and 1,3,5-trimethylbenzene. The presence of an isopropyl group in (1-methylethyl)benzene, which provides a higher steric hindrance than in the case of 1,3,5-trimethylbenzene, increases the $\gamma_{13}^{\infty }$ value nearly six time. By increasing the number of dislocated π electrons to six (as in the case of aromatic compounds), a further increase of ionic liquid-solute interactions can be achieved. At the same time, a strong influence of alkyl substituents in the ring is observed; i.e., for both ionic liquids, the values of $\gamma_{13}^{\infty }$ increase in the following sequence: benzene < toluene < xylene < 1,3,5-trimethylbenzene. In the case of heteroatom-containing compounds, such as alcohols, aldehydes and ketones, there is a significant increase in interaction force between these compounds and the studied ionic liquids. Additionally, a trend is observed; namely, that the interaction force decreases with increasing length of the alkyl chain. Particularly strong solute-solvent interactions are indicated by the values of $\gamma_{13}^{\infty }$ below 1; such values were determined for the compounds containing heteroatoms with high electronegativity, e.g., oxygen and chlorine atoms. In the case of [C_8_C_1_Mor][TFSI], only alcohols with CH_3_(CH_2_)_3_− and longer-chain alcohols are characterized by $\gamma_{13}^{\infty }$ values greater than 1. At the same time, it is noticeable that the presence of a long substituent in the morpholine ring has a stronger differentiating effect on the interactions with the compounds, displaying different chemical characteristics. In the case of [C_2_C_1_Mor]^+^ cation, based on the slopes of relation of ln$\gamma_{13}^{\infty }$ in function of inversion of temperature, for *n*-alkane, *n*-alkene, *n*-alkyne, alcohol and aldehyde, any significant difference can be observed. Figure 2 compares the relative difference of $\gamma_{13}^{\infty }$ for both studied ILs. The [C_2_C_1_Mor][TFSI] should be treated as 100% (reference level). It can be found that for all investigated compounds, the $\gamma_{13}^{\infty }$ values determined for the ionic liquid containing [C_2_C_1_Mor]^+^ cation are lower than those for the ionic liquid containing [C_2_C_1_Mor]^+^ cation. At the same time, it was found that polar compounds showed the lowest decrease in the $\gamma_{13}^{\infty }$ value, while in the case of *n*-alkanes, those values were at least four times lower for [C_8_C_1_Mor]^+^ compared to [C_2_C_1_Mor]^+^. Nevertheless, the shape of the curve indicates that the alkyl parts of solutes have significant influence on the overall interactions, which suppress, to a certain extent, the effect of the polar group. It is noticeable that, with increasing length of the alkyl chain in solutes, a disproportion between the values obtained for both ionic liquids increases.

The values of activity coefficients at infinite dilution obtained for selected solutes in this study, together with literature data for ILs containing the same anion and different cations, are listed in Table 3. 

The ionic liquids selected for comparative purposes consist of cations, which contain analogous substituents and different types of carbon rings. When analyzing the data, one can notice that in the case of cations containing an alkyl chain of eight carbon atoms, the effect of the ring-type on the $\gamma_{13}^{\infty }$ values is insignificant. Only in the case of alcohols, when the formation of a hydrogen bond with the oxygen atom present in the morpholine ring is possible, the values of $\gamma_{13}^{\infty }$ become significantly smaller, which indicates strong interactions with the ionic liquid. Decreasing the length of the alkyl substituent brings out the properties of the ring present in the cation. As a consequence, significant differences in the values of $\gamma_{13}^{\infty }$ were noted for almost all compounds being compared. The lowered $\gamma_{13}^{\infty }$ value for ethanol in comparison to other liquids can be considered as distinctive.

The excess partial molar enthalpies at infinite dilution of the studied solutes were determined from the relationship between ln$\gamma_{13}^{\infty }$ and T^−1^ based on the Gibbs-Helmholtz equation. The results obtained for both ILs and the associated coefficients of determination are listed in Table 4 and Table 5. 

For the two investigated ionic liquids, similar patterns emerged with regards to alkanes, alkenes, alkynes and alcohols. For all aforementioned solutes, the values of $H_{1}^{E,\infty }$ were positive, which indicates that the $\gamma_{13}^{\infty }$ values decrease with decreasing temperatures, but these changes are relatively small. For aromatic compounds and the most polar compounds containing heteroatoms (except for alcohols), the obtained values of $H_{1}^{E,\infty }$ were negative, which points to stronger solute-solvent interactions compared to solute-solute interactions. For most investigated compounds dissolved in both ionic liquids, the values of $H_{1}^{E,\infty }$ had the same sign. Only in the cases of benzene and acetonitrile did the obtained values have different signs, i.e., positive for [C_8_C_1_Mor]^+^ and negative for [C_2_C_1_Mor]^+^.

High negative values of excess molar entropy, calculated by using the van ‘t Hoff equation for alcohols in both ionic liquids, could have resulted from the formation of hydrogen bonds with oxygen present in the morpholine ring.

The values of selectivity, *S_ij_* at 323.15 K for the hexane/benzene system, calculated by using Equation (5), are presented in Table 6. For comparative purposes, the corresponding values for [C_2_C_1_IM]^+^, [C_8_C_1_IM]^+^, [C_2_py]^+^ and most commonly used solvents such as sulfolane and *N*-methyl-2-pirrolidone [NMP] are also listed in Table 6.

It is noticeable that the elongation of the alkyl chain present in the morpholinium ring resulted in a significant drop in selectivity of [C_8_C_1_Mor]^+^, in comparison to [C_2_C_1_Mor]^+^. The separation efficiency of [C_8_C_1_Mor]^+^ is comparable to that of [C_8_C_1_IM]^+^; i.e., it is listed as the second ionic liquid with an alkyl chain of eight carbon atoms, while at the same time, its separation efficiency is lower than those of other selected ILs. The selectivity of [C_2_C_1_Mor][TFSI] is lower than those of [C_2_C_1_IM][TFSI] and [C_2_Pyr][TFSI], the latter two containing aromatic systems. The separation efficiency of [C_2_C_1_Mor][TFSI] in the hexane/benzene system is higher than that displayed by the solvents most commonly used in practice.

## 4. Materials and Methods 

The ionic liquids based on *N*-alkyl-*N*-methylmorpholinium cations, *N*-ethyl-*N*-methylmorpholinium bis(trifluoromethanesulfonyl)imide, [C_2_C_1_Mor][TFSI] and *N*-methyl-*N*-octylmorpholinium bis(trifluoromethanesulfonyl)imide, [C_8_C_1_Mor][TFSI] were synthesized and purified according to the procedure described in our previous paper [29]. The provenance and mass fraction purity of the studied ionic liquids are presented in Table S1 (see Supplementary Material). Columns (1 m long, with an inner diameter of 2.1 mm) were made out of stainless steel tubing (304 grade) purchased from Supelco (Bellefonte, PA, USA). Prior to use, the columns were cleaned by sequential washing with a detergent solution, methanol and deionized water (Milli-Q ultra-pure water system, Burlington, MA, USA). Chromosorb W HP-DMCS 80/100 mesh (Sigma-Aldrich, Hamburg, Germany) was used as a solid support. In order to achieve a homogeneous distribution of ionic liquids on the solid support, HPLC-grade dichloromethane was applied as solvent. Coating solid support with the ionic liquid was performed by dispersing a weighted amount of solid support in a solution of the ILs in dichloromethane, followed by gradual evaporation of the solvent using a rotary evaporator. The parameters of the evaporation process (thermostat temperature and rotational speed) were chosen to enable a uniform distribution of ionic liquids on the solid support surface (~10 h). In the last stage, prepared column packing was dried under vacuum conditions to remove the residues of dichloromethane and present volatile organic compounds. The mass of ionic liquid, support and ionic liquid-coated support was gravimetrically controlled with an accuracy of 0.0001 g. The prepared column packing was introduced into the column in portions to obtain the maximal packing of the column. The packed column was conditioned by blowing a carrier gas for 12 h (N_2_, 10^−5^ m^3^·min^−1^) at temperature 373.15 K. In addition, the retention times of benzene and hexane were measured after each 48 h to confirm that the column properties have not been changing during the entire measuring cycle. In order to evaluate the influence of the amount of ionic liquid on the determined values of $\gamma_{13}^{\infty }$, two columns with different mass fractions of the ionic liquid, namely, 40 *w*% and 44 *w*%, were prepared for each ionic liquid, i.e., [C_2_C_1_Mor][TFSI] and [C_8_C_1_Mor][TFSI]. The difference in results obtained for both column loadings was less than 3% for [C_2_C_1_Mor][TFSI] and 2.5% for [C_8_C_1_Mor][TFSI]. Experiments were carried out at six different temperatures, i.e., T= 313.15, 323.15, 333.15, 343.15, 353.15 and 363.15 K. The column temperature was controlled with an accuracy of ±0.02 K.

The inverse gas chromatography technique was used in this work for the measurements of activity coefficients at infinite dilution ($\gamma_{13}^{\infty }$). All analyses were carried out on a Agilent system that consists of an Agilent 7890 A gas chromatograph coupled with a flame ionization detector (Palo Alto, CA, USA). Data were collected and processed with the use of Agilent Software (Palo Alto, CA, USA). Nitrogen (99.999%) was used as carrier gas. Solute samples (1 μL) were introduced into GC injector port. In order to ensure the infinite dilution of the sample inside the column, the injector worked in split mode in the range from 1:10 to 1:50, depending on the detector response to solute. Prior to a series of measurements, the column was thermostated at a given temperature for at least 30 min. Each measurement was repeated 3 times to ensure the reproducibility of the obtained results. The retention time for each solute was obtained from the difference between the detected time for solute and the time of the nonretained component (methane). Obtained times were generally reproducible within 0.001–0.05 min. The differences depend on type of the individual solute and on the temperature. The flow rate of the carrier gas was measured by means of automatic soap bubble flow meter connected to the GC detector outlet with uncertainty of 1 × 10^−7^ m^3^·min^−1^. The flow rate of carrier gas was corrected for water vapor pressure. The measured pressure in the column varied between 10 and 50 kPa, depending on the temperature and the flow rate of carrier gas. The inlet and outlet pressure (the outlet pressure is equal to atmospheric pressure) were measured by means of internal chromatograph pressure controller (with uncertainty of ±0.1 kPa). 

## 5. Conclusions

In this paper, the interactions of 48 organic compounds with two ionic liquids based on bis(trifluoromethanesulfonyl)imide anion and [C_2_C_1_Mor]^+^ and [C_8_C_1_Mor]^+^ cations are described. The studied organic solutes represented different compound types, i.e., *n*-alkanes, *n*-alkenes, *n*-alkynes, alcohols, ketones, aromatic compounds and other polar solvents. The two ILs differed only in the chain length of alkyl substituent. The values of activity coefficient at infinite dilution at temperature range 313.15–363.15 K were determined by using gas-liquid chromatography. Based on the relationship between ln$\gamma_{13}^{\infty }$ and T^−1^, the values of excess partial molar enthalpy and excess entropy at infinite dilution were calculated. Also, the values of selectivity at infinite dilution were determined for both ionic liquids in the hexane/benzene system. It was demonstrated that separation efficiency in this system decreases as the number of carbons in the alkyl substituent increases. The obtained S_ij_ values for the ionic liquid containing [C_2_C_1_Mor]^+^ cation were higher than those of conventional solvents such as NMP and sulfolane. 

## Figures and Tables

**Table 1:** The experimental activity coefficients at infinite dilution $\gamma_{13}^{\infty }$ for the solutes in ionic liquid [C_2_C_1_Mor][TFSI] (313.15–363.15 K).

Solute	T\K					
	313.15	323.15	333.15	343.15	353.15	363.15
*n*-pentane	18.400	16.900	16.000	14.400	13.500	12.300
*n*-hexane	28.900	25.400	23.900	21.800	20.400	18.400
*n*-heptane	40.600	37.300	33.100	29.700	26.900	24.900
*n*-octane	60.300	52.400	46.900	41.800	38.400	33.800
*n*-nonane	77.700	65.900	58.400	51.200	45.200	40.400
*n*-decane	96.600	83.400	70.900	61.300	54.400	47.400
cyclohexane	17.800	16.400	15.100	13.900	12.800	11.600
1-hexene	6.570	6.050	5.690	5.260	5.000	4.810
1-heptene	9.790	8.980	8.430	7.810	7.320	6.980
1-octene	14.400	13.100	12.100	11.000	10.100	9.520
1-nonene	18.600	16.900	15.200	14.000	12.800	11.900
1-decene	23.600	21.300	19.000	17.000	15.400	14.100
1-hexyne	3.530	3.380	3.230	3.100	3.020	2.910
1-heptyne	4.880	4.580	4.420	4.210	4.040	3.910
1-octyne	6.920	6.610	6.270	5.960	5.610	5.340
1-nonyne	9.470	8.930	8.410	7.880	7.490	7.110
1-decyne	12.000	11.200	10.500	9.880	9.360	8.790
methanol	0.992	0.933	0.854	0.826	0.768	0.727
ethanol	1.370	1.290	1.200	1.140	1.080	1.010
propanol	1.830	1.700	1.600	1.490	1.390	1.300
propan-2-ol	1.720	1.620	1.500	1.450	1.360	1.300
butan-1-ol	2.880	2.610	2.390	2.210	2.060	1.920
tert-butanol	1.900	1.800	1.690	1.610	1.540	1.460
pentanol	3.870	3.490	3.160	2.910	2.680	2.460
2-methylopentan-2-ol	4.120	3.760	3.330	3.040	2.840	2.570
hexanol	4.950	4.370	3.910	3.580	3.250	2.950
octanol	11.000	9.580	8.290	7.190	6.470	5.720
benzene	1.200	1.209	1.219	1.225	1.235	1.242
toluene	1.650	1.700	1.730	1.760	1.810	1.840
*p*-xylene	2.490	2.560	2.630	2.700	2.810	2.870
(1-methylethyl) benzene	12.200	11.000	9.840	8.880	8.120	7.390
1,3,5-trimethylbenzene	3.460	3.580	3.670	3.740	3.820	3.920
acetaldehyde	0.350	0.363	0.374	0.389	0.403	0.413
propionaldehyde	0.506	0.523	0.534	0.552	0.570	0.585
butyraldehyde	0.741	0.757	0.777	0.796	0.815	0.830
valeryaldehyde	1.192	1.214	1.233	1.262	1.286	1.306
capronaldehyde	1.810	1.847	1.865	1.891	1.911	1.934
pentan-2-one	0.708	0.741	0.776	0.813	0.851	0.883
pentan-3-one	0.773	0.817	0.860	0.894	0.942	0.980
cyclopentanone	0.481	0.495	0.510	0.526	0.544	0.558
cyclohexanone	-	0.832	0.866	0.896	0.932	0.961
ethyl acetate	0.832	0.870	0.897	0.936	0.980	1.020
tetrahydrofurane	0.685	0.710	0.734	0.758	0.790	0.813
1,4-dioxane	0.437	0.464	0.488	0.520	0.554	0.579
chlorobenzene	2.120	2.160	2.186	2.214	2.244	2.273
chloroform	1.140	1.180	1.221	1.263	1.292	1.330
acetonitrile	0.333	0.340	0.345	0.350	0.357	0.361
pyridine	0.473	0.497	0.523	0.539	0.570	0.593

Standard uncertainties u are u($\gamma_{13}^{\infty }$) < 4%; u(T) = 0.02 K.

**Table 2:** The experimental activity coefficients at infinite dilution $\gamma_{13}^{\infty }$ for the solutes in ionic liquid [C_8_C_1_Mor][TFSI] (313.15–363.15 K).

Solute	T\K					
	313.15	323.15	333.15	343.15	353.15	363.15
*n*-pentane	4.77	4.40	4.18	3.97	3.79	3.66
*n*-hexane	5.56	5.29	4.99	4.71	4.46	4.23
*n*-heptane	7.03	6.58	6.15	5.78	5.46	5.09
*n*-octane	8.34	7.68	7.06	6.62	6.17	5.79
*n*-nonane	10.1	9.26	8.49	7.77	7.19	6.66
*n*-decane	12.3	11.3	10.3	9.27	8.43	7.69
cyclohexane	3.83	3.53	3.35	3.15	2.99	2.87
1-hexene	4.07	3.81	3.61	3.40	3.20	3.06
1-heptene	5.25	4.89	4.51	4.32	4.03	3.79
1-octene	6.64	6.21	5.68	5.26	4.97	4.73
1-nonene	7.53	6.89	6.45	5.85	5.52	5.19
1-decene	8.85	8.05	7.42	6.78	6.29	5.88
1-hexyne	2.48	2.37	2.27	2.21	2.17	2.12
1-heptyne	3.04	2.91	2.82	2.73	2.61	2.52
1-octyne	4.01	3.78	3.63	3.46	3.36	3.23
1-nonyne	5.05	4.73	4.53	4.32	4.13	3.99
1-decyne	6.05	5.71	5.39	5.16	4.94	4.73
methanol	0.739	0.689	0.648	0.616	0.587	0.555
ethanol	0.849	0.786	0.737	0.694	0.652	0.617
propanol	0.934	0.871	0.800	0.756	0.707	0.663
propan-2-ol	0.912	0.835	0.782	0.738	0.692	0.654
butan-1-ol	1.09	1.00	0.928	0.865	0.815	0.763
tert-butanol	0.890	0.827	0.763	0.708	0.654	0.612
pentanol	1.38	1.28	1.18	1.09	1.01	0.947
2-methylopentan-2-ol	1.31	1.20	1.11	1.04	0.975	0.914
hexanol	1.64	1.50	1.39	1.29	1.20	1.13
octanol	2.42	2.19	2.02	1.85	1.71	1.58
benzene	0.604	0.589	0.573	0.570	0.560	0.547
toluene	0.666	0.685	0.693	0.704	0.716	0.726
*p*-xylene	0.838	0.890	0.892	0.927	0.967	0.994
(1-methylethyl) benzene	6.98	6.06	5.40	4.86	4.32	3.87
1,3,5-trimethylbenzene	1.08	1.10	1.14	1.14	1.18	1.20
acetaldehyde	0.257	0.260	0.264	0.266	0.267	0.270
propionaldehyde	0.286	0.293	0.298	0.303	0.307	0.309
butyraldehyde	0.334	0.341	0.345	0.355	0.359	0.366
valeryaldehyde	0.408	0.420	0.428	0.436	0.442	0.451
capronaldehyde	0.489	0.503	0.517	0.530	0.541	0.555
pentan-2-one	0.255	0.269	0.282	0.295	0.306	0.321
pentan-3-one	0.193	0.201	0.213	0.225	0.234	0.244
cyclopentanone	-	0.218	0.228	0.236	0.243	0.252
cyclohexanone	-	-	0.401	0.417	0.432	0.447
ethyl acetate	0.331	0.343	0.356	0.370	0.380	0.394
tetrahydrofurane	0.284	0.294	0.302	0.311	0.318	0.326
1,4-dioxane	0.303	0.310	0.320	0.327	0.331	0.337
chlorobenzene	0.731	0.744	0.759	0.772	0.784	0.797
chloroform	0.559	0.575	0.590	0.603	0.619	0.629
acetonitrile	0.285	0.281	0.277	0.275	0.270	0.266
pyridine	0.242	0.246	0.249	0.253	0.254	0.257

Standard uncertainties u are u($\gamma_{13}^{\infty }$) < 4%; u(T) = 0.02 K.

**Table 3:** The values of $\gamma_{13}^{\infty }$ for selected solutes in different ILs (T = 323.15 K).

Solute	$\gamma_{13}^{\infty }$					
	[C_2_C_1_Mor] [TFSI]	[C_8_C_1_Mor] [TFSI]	[C_2_C_1_IM] [TFSI] ^a^	[C_8_C_1_IM] [TFSI] ^a^	[C_2_Pyr] [TFSI] ^a^	[C_8_Pyr] [TFSI] ^a^
hexane	25.4	5.3	26.3	5.32	33.5	4.92
cyclohexane	16.4	3.5	9.43	3.64	18.5	
hexene	6.0	3.8	8.87	3.29	14.6	3.19
ethanol	1.3	0.79	1.93	1.46	1.89	
benzene	1.2	0.59	0.72	0.63	1.26	
toluene	1.7	0.69	1.81	0.82	1.86	

^a^ Reference [11].

**Table 4:** Limiting partial molar excess Gibbs frees energies $\Delta G_{1}^{E,\infty }$, enthalpies, $\Delta H_{1}^{E,\infty }$ and entropies; $T\Delta S_{1}^{E,\infty }$ for the solutes in ionic liquid [C_2_C_1_Mor][TFSI] at the reference temperature T_ref_ = 323.15 K.

Solute	*R* ^2^	$\Delta G_{1}^{E,\infty }$ kJ·mol^−1^	$\Delta H_{1}^{E,\infty }$ kJ·mol^−1^	$T\Delta S_{1}^{E,\infty }$ kJ·mol^−1^
*n*-pentane	0.9925	7.5	7.6	0.05
*n*-hexane	0.9922	7.5	8.1	0.61
*n*-heptane	0.9973	9.4	9.5	0.16
*n*-octane	0.9979	10.7	10.7	0.00
*n*-nonane	0.9992	13.2	12.2	−1.0
*n*-decane	0.9994	15.1	13.5	−1.6
cyclohexane	0.9945	8.5	8.0	−0.48
1-hexene	0.9938	7.1	6.0	−1.1
1-heptene	0.9977	7.0	6.4	−0.53
1-octene	0.9985	9.0	7.9	−1.0
1-nonene	0.9995	9.3	8.5	−0.88
1-decene	0.9991	11.6	9.9	−1.7
1-hexyne	0.9967	4.1	3.7	−0.40
1-heptyne	0.9964	4.2	4.2	−0.06
1-octyne	0.9947	4.8	4.9	0.12
1-nonyne	0.9991	5.1	5.5	0.40
1-decyne	0.9992	5.2	5.9	0.63
methanol	0.9929	12.0	5.9	−6.1
ethanol	0.9967	10.8	5.8	−5.1
propanol	0.9973	11.6	6.5	−5.1
propanol	0.9959	9.4	5.3	−4.0
1-butanol	0.9994	12.7	7.7	−5.1
tert-butanol	0.9986	8.3	4.9	−3.4
pentanol	0.9996	13.6	8.5	−5.1
2-methylopentan-2-ol	0.9970	14.3	8.9	−5.4
hexanol	0.9991	15.3	9.6	−5.6
octanol	0.9981	18.5	12.3	−6.2
benzene	0.9972	−1.8	−0.6	1.2
toluene	0.9896	−5.4	−2.0	3.4
*p*-xylene	0.9909	−8.0	−2.7	5.3
(1-methylethyl) benzene	0.9995	12.7	9.6	−3.1
1,3,5-trimethylbenzene	0.9949	−8.0	−2.3	5.7
acetaldehyde	0.9971	−3.6	−3.2	0.5
propionaldehyde	0.9931	−3.7	−2.7	1.0
butyraldehyde	0.9981	−3.7	−2.2	1.5
valeryaldehyde	0.9947	−4.0	−1.8	2.3
capronaldehyde	0.9934	−4.1	−1.2	2.9
pentan-2-one	0.9992	−7.7	−4.3	3.5
pentan-3-one	0.9990	−8.4	−4.5	3.9
cyclopentanone	0.9980	−3.8	−2.8	1.0
cyclohexanone	0.9992	−6.6	−3.5	3.0
ethyl acetate	0.9941	−7.2	−3.8	3.4
tetrahydrofurane	0.9970	−5.6	−3.3	2.3
1,4-dioxane	0.9970	−8.8	−5.4	3.3
chlorobenzene	0.9966	−4.6	−1.2	3.3
chloroform	0.9977	−6.3	−2.9	3.4
acetonitrile	0.9970	−0.12	−1.5	−1.4
pyridine	0.9955	−6.6	−4.3	2.4

**Table 5:** Limiting partial molar excess Gibbs frees energies $\Delta G_{1}^{E,\infty }$, enthalpies, $\Delta H_{1}^{E,\infty }$ and entropies; $T\Delta S_{1}^{E,\infty }$ for the solutes in ionic liquid [C_2_C_1_Mor][TFSI] at the reference temperature T_ref_ = 323.15 K.

Solute	*R* ^2^	$\Delta G_{1}^{E,\infty }$ kJ·mol^−1^	$\Delta H_{1}^{E,\infty }$ kJ·mol^−1^	$T\Delta S_{1}^{E,\infty }$ kJ·mol^−1^
pentane	0.9914	5.9	4.96	−1
hexane	0.9975	6	5.21	−0.74
heptane	0.9981	7	6.02	−1
octane	0.9995	8.3	6.88	−1.4
nonane	0.9991	9.7	7.84	−1.9
decane	0.9975	11.5	8.99	−2.5
cyclohexane	0.9962	7.5	5.47	−2.1
hex-1-ene	0.9990	7.3	5.46	−1.9
hept-1-ene	0.9969	7.9	6.09	−1.8
oct-1-ene	0.9954	8.3	6.61	−1.7
non-1-ene	0.9971	9	7.09	−1.9
dec-1-ene	0.9996	10	7.79	−2.2
hex-1-yne	0.9809	3.5	2.89	−0.57
hept-1-yne	0.9951	4.1	3.49	−0.61
oct-1-yne	0.9965	4.5	4.04	−0.45
non-1-yne	0.9971	4.6	4.40	−0.21
dec-1-yne	0.9991	4.6	4.65	0.032
methanol	0.9986	11.6	5.31	−6.3
ethanol	0.9997	12.6	5.99	−6.6
propanol	0.9988	13.3	6.48	−6.9
propan-2-ol	0.9982	12.8	6.17	−6.6
butan-1-ol	0.9995	13.2	6.62	−6.6
tert-butanol	0.9988	14.8	7.16	−7.7
pentanol	0.9991	13.8	7.23	−6.6
2-methylopentan-2-ol	0.9992	12.9	6.72	−6.2
hexanol	0.9994	12.9	7.01	−5.9
octanol	0.9994	13.9	7.99	−5.9
benzene	0.9810	5	1.79	−3.2
toluene	0.9897	−2.1	−1.56	0.53
*p*-xylene	0.9665	−5.8	−3.08	2.7
(1-methylethyl) benzene	0.9992	17.1	10.97	−6.1
1.3.5-trimethylbenzene	0.9792	−4.1	−1.94	2.2
acetaldehyde	0.9706	1.8	−0.91	−2.7
propionaldehyde	0.9863	0.36	−1.47	−1.8
butyraldehyde	0.9929	−0.59	−1.74	−1.2
valeryaldehyde	0.9952	−1.3	−1.82	−0.52
capronaldehyde	0.999	−2.9	−2.37	0.52
pentan-2-one	0.9991	−5	−4.25	0.72
pentan-3-one	0.9983	−4.9	−4.61	0.32
cyclopentanone	0.9978	−2.8	−3.44	−0.64
cyclohexanone	0.9995	−4.7	−3.61	1
ethyl acetate	0.9987	−3.7	−3.31	0.44
tetrahydrofurane	0.9991	−1.9	−2.60	−0.69
1,4-dioxane	0.9911	−0.92	−2.03	−1.1
chlorobenzene	0.9996	−2.5	−1.63	0.84
chloroform	0.9985	−3	−2.26	0.78
acetonitrile	0.9883	6	1.30	−4.7
pyridine	0.9935	1.5	−1.16	−2.6

**Table 6:** Selectivity. *S_ij_* for hexane/benzene and hexane/cyclohexane separation at T = 323.15 K for selected ionic liquids based on [TFSI]^−^ anion.

*S_ij_*	[C_2_C_1_Mor] [TFSI]	[C_8_C_1_Mor] [TFSI]	[C_2_C_1_IM] [TFSI] ^a^	[C_8_C_1_IM] [TFSI] ^a^	[C_2_Pyr] [TFSI] ^a^	[NMP] ^a^	Sulfolane ^a^
hexane/benzene	21.02	8.98	36.53	8.44	26.59	13.20	20.80
hexane/cyclohexane	1.55	1.50	2.79	1.46	1.81	8.10	10.20

^a^ Reference [11].

## Supplementary Materials

The following are available online, Table S1: Provenance and mass fraction purity of the ionic liquids studied. Table S2: Critical constants. Vc, Tc., Pc and ω of the solutes and the carrier gas used in the calculation of the virial coefficients. Table S3: Molar volume vapor pressure and virial coefficients: B11 and B12 used in the calculation of at temperatures 312.15 to 363.15 K for ionic liquids [Mor1,2][TFSI] and [Mor1,8][TFSI].
