# Supplementary material for: Measurements of Activity Coefficients at Infinite Dilution for Organic Solutes in the Ionic Liquids N-Ethyl- and N-Octyl-N-methylmorpholinium Bis(trifluoromethanesulfonyl)imide. A Useful Tool for Solvent Selection
Source: Molecules. 2020 Feb 1;25(3):634. doi: 10.3390/molecules25030634 (PMC7037026; doi:10.3390/molecules25030634)
Supplement: Supplementary file 1 [file molecules-25-00634-s001.pdf]

SUPPLEMENTARY MATERIAL

Measurements of activity coefficients at infinite dilution for organic solutes in the ionic liquids *N*-ethyl- and *N*-octyl-*N*-methylmorpholinium bis(trifluoromethanesulfonyl)imide. A useful tool for solvent selection.

Łukasz Marcinkowski [1,], Joachim Eichenlaub [1], Elham Ghasemi [2], Żaneta Polkowska [2] and Adam Kloskowski [1]*

[1] Department of Physical Chemistry, Faculty of Chemistry, Gdansk University of Technology, Narutowicza Str.11/12, Gdansk 80-233 Poland
[2] Department of Analytical Chemistry, Faculty of Chemistry, Gdansk University of Technology, Narutowicza Str.11/12, Gdansk 80-233 Poland
* Correspondence: lukasz.marcinkowski@pg.edu.pl (*Ł. Marcinkowski*)

Table S1. Provenance and mass fraction purity of the ionic liquids studied.

Chemical name	Source	CAS number	Mass fraction purity (%)	Water content [c] (%mass)	Bromide content [d] (%mass)
$[Mor_{1,2}][TFSI]$	Present work [a]	706785-83-3	>99% [b]	0.017%	0.0065%
$[Mor_{1,8}][TFSI]$	Present work [a]	-	>99% [b]	0.020%	0.0055%

[a] synthesized in our laboratory
[b] from ^{1}H NMR and ^{13}C NMR spectroscopic measurements
[c] Karl-Fischer titration method
[d] from Ionic Chromatography analysis

Table S2: Critical constants. V_c , T_c. , P_c and ω of the solutes and the carrier gas used in the calculation of the virial coefficients.

**Solute**	**T_c**	**P_c**	**V_c**	**ω**
	[K]	[kPa]	[cm^3•mol^{-1}]	
n-pentane	469.70	3370	311.00	0.251
n-hexane	507.60	3025	368.00	0.299
cyclohexane	553.80	4080	308.00	0.208
n-heptane	540.20	2740	428.00	0.350
n-octane	568.70	2490	492.00	0.397
n-nonane	594.60	2290	555.00	0.443
n-decane	617.70	2110	624.00	0.490
1-hexene	504.00	3210	355.00	0.286
1-heptene	537.30	2920	409.00	0.344
1-octene	567.00	2680	468.00	0.392
1-nonene	594.00	2330	526.00	0.410
1-decene	617.00	2220	584.00	0.475
1-hexyne	516.00	3620	322.00	0.333
1-heptyne	559.00	3140	386.00	0.273
1-octyne	585.00	2820	441.00	0.323

1-nonyne	598.05	2610	497.00	0.471
1-decyne	619.85	2370	552.00	0.518
methanol	512.64	8092	113.02	0.564
ethanol	513.92	6132	168.00	0.649
propanol	536.78	5168	218.00	0.620
2-propanol	604.00	4530	233.00	0.539
1-butanol	563.05	4424	274.00	0.591
tert-butanol	506.21	3973	275.00	0.590
pentanol	588.15	3909	326.00	0.579
2-methylo-2-pentanol	559.50	3470	380.00	0.573
hexanol	610.30	3417	387.00	0.576
octanol	652.50	2860	490.00	0.594
acetaldehyde	466.00	5550	154.00	0.291
propionaldehyde	504.40	5260	223.00	0.298
butyraldehyde	537.20	4320	258.00	0.277
valeryaldehyde	566.10	3970	333.00	0.422
capronaldehyde	591.00	3460	388.00	0.459
2-pentanone	561.10	3690	301.00	0.346
3-pentanone	561.50	3730	336.00	0.342
cyclopentanone	624.50	4600	268.00	0.288
cyclohexanone	653.00	4000	311.00	0.299
1.4-dioxane	587.00	5170	238.00	0.281
tetrahydrofurane	540.20	5190	224.00	0.226
ethyl acetate	523.20	3830	286.00	0.361
benzene	562.05	4895	256.00	0.209
toluene	591.75	4108	316.00	0.261
p-xylene	616.20	3511	378.00	0.322
(1-methylethyl)benzene	631.00	3209	434.70	0.326
1.3.5-trimethylbenzene	637.30	3127	430.00	0.399
chlorobenzene	632.40	4520	308.00	0.251
chloroform	536.50	5500	240.00	0.257
acetonitrile	545.50	4890	175.00	0.342
pyridine	620.00	5670	254.00	0.242
nitrogen	126.20	3350	89.20	0.037

Table S3: Molar volume V_1^* vapor Pressure P_1^* and virial coefficients: B_{11} and B_{12} used in the calculation of γ_{13}^* at temperatures 312.15 to 363.15 K for ionic liquids [$Mor_{1,2}$][TFSI] and [$Mor_{1,8}$][TFSI].

T	**V_1***	**P_1***	**B_{11}**	**B_{12}**
K	cm^3•mol^{-1}	kPa	cm^3•mol^{-1}	cm^3•mol^{-1}
		n-pentane		
313.15	119.363	115.854	-1171.158	-103.673
323.15	121.505	160.284	-986.799	-95.457
333.15	123.797	216.985	-912.255	-87.883
343.15	126.259	288.035	-846.632	-80.878
353.15	128.918	375.613	-788.403	-74.382
363.15	131.808	481.971	-736.373	-68.341
		n-hexane		
313.15	134.181	36.673	-1863.550	-120.827
323.15	136.180	53.499	-1535.522	-111.255
333.15	138.291	76.020	-1406.742	-102.443
343.15	140.527	105.500	-1295.280	-94.304
353.15	142.901	143.324	-1197.893	-86.766
363.15	145.432	190.986	-1112.086	-79.764
		cyclohexane		
313.15	110.829	23.708	-1771.190	-126.680
323.15	112.201	35.279	-1450.290	-117.459
333.15	113.634	51.022	-1326.550	-108.965
343.15	115.133	71.927	-1220.490	-101.117
353.15	116.705	99.094	-1128.630	-93.845
363.15	118.356	133.719	-1048.280	-87.088
		n-heptane		
313.15	149.564	12.103	-2861.655	-136.417
323.15	151.555	18.569	-2301.553	-125.522
333.15	153.642	27.641	-2087.435	-115.505
343.15	155.833	40.047	-1905.013	-106.266
353.15	158.139	56.621	-1747.949	-97.717
363.15	160.572	78.306	-1611.426	-89.786
		n-octane		
313.15	165.977	4.282	-4259.578	-150.979
323.15	167.914	6.855	-3346.495	-138.780
333.15	169.933	10.619	-3005.103	-127.578
343.15	172.041	15.970	-2718.160	-117.257
353.15	174.246	23.385	-2474.258	-107.717
363.15	176.557	33.426	-2264.805	-98.873
		n-nonane		
313.15	182.114	1.537	-6169.619	-164.481
323.15	184.177	2.579	-4738.902	-151.019
333.15	186.319	4.173	-4213.897	-138.669
343.15	188.547	6.533	-3777.769	-127.302
353.15	190.866	9.930	-3411.232	-116.804
363.15	193.284	14.693	-3099.858	-107.082
		n-decane		

313.15	198.219	0.546	-8760.922	-176.769
323.15	200.316	0.966	-6590.240	-162.059
333.15	202.488	1.641	-5805.845	-148.578
343.15	204.739	2.685	-5160.610	-136.179
353.15	207.076	4.251	-4623.544	-124.740
363.15	209.504	6.530	-4171.575	-114.153
		1-hexene		
313.150	128.993	45.058	-1523.577	-116.939
323.150	130.996	64.660	-1390.078	-107.726
333.150	133.112	90.579	-1275.463	-99.242
343.150	135.355	124.156	-1176.033	-91.404
353.150	137.740	166.854	-1088.974	-84.143
363.150	140.287	220.252	-1012.113	-77.398
		1-heptene		
313.150	144.380	14.999	-2326.637	-132.293
323.150	146.280	22.629	-2097.629	-121.779
333.150	148.271	33.191	-1904.478	-112.112
343.150	150.364	47.465	-1739.674	-103.196
353.150	152.567	66.340	-1597.580	-94.945
363.150	154.894	90.813	-1473.909	-87.290
		1-octene		
313.150	160.703	5.149	-3421.745	-146.584
323.150	162.698	8.170	-3049.847	-134.817
333.150	164.779	12.548	-2740.788	-124.013
343.150	166.952	18.720	-2480.789	-114.057
353.150	169.226	27.205	-2259.598	-104.856
363.150	171.610	38.609	-2069.491	-96.327
		1-nonene		
313.150	177.008	1.790	-5051.313	-165.726
323.150	179.018	2.994	-4460.386	-152.412
333.150	181.106	4.826	-3974.866	-140.194
343.150	183.278	7.523	-3570.893	-128.942
353.150	185.539	11.384	-3230.831	-118.547
363.150	187.898	16.769	-2941.478	-108.916
		1-decene		
313.150	193.267	0.645	-7008.931	-175.414
323.150	195.314	1.133	-6119.540	-161.019
333.150	197.433	1.911	-5396.613	-147.828
343.150	199.630	3.105	-4801.512	-135.696
353.150	201.911	4.883	-4305.811	-124.502
363.150	204.281	7.455	-3888.342	-114.143
		1-hexyne		
313.15	117.711	32.693	-1560.770	-115.693
323.15	119.369	48.186	-1415.450	-106.593
333.15	121.115	69.188	-1291.717	-98.226

343.15	122.958	97.027	-1185.208	-90.508
353.15	124.910	133.194	-1092.625	-83.367
363.15	126.984	179.330	-1011.440	-76.741
		1-heptyne		
313.15	134.516	11.617	-2359.482	-140.047
323.15	136.209	17.864	-2127.290	-129.445
333.15	137.976	26.625	-1932.244	-119.690
343.15	139.823	38.586	-1766.407	-110.685
353.15	141.759	54.532	-1623.851	-102.347
363.15	143.790	75.342	-1500.085	-94.606
		1-octyne		
313.15	150.983	3.863	-3457.531	-154.699
323.15	152.794	6.293	-3081.014	-142.825
333.15	154.679	9.884	-2769.190	-131.912
343.15	156.646	15.028	-2507.670	-121.850
353.15	158.700	22.194	-2285.787	-112.544
363.15	160.851	31.933	-2095.528	-103.912
		1-nonyne		
313.15	167.773	0.936	-5026.806	-159.136
323.15	169.567	1.679	-4416.674	-146.183
333.15	171.427	2.887	-3917.300	-134.314
343.15	173.356	4.777	-3503.416	-123.400
353.15	175.360	7.639	-3156.368	-113.331
363.15	177.445	11.845	-2862.214	-104.015
		1-decyne		
313.15	184.107	0.496	-7087.601	-172.401
323.15	185.962	0.884	-6168.513	-158.156
333.15	187.880	1.513	-5423.112	-145.117
343.15	189.865	2.496	-4810.913	-133.139
353.15	191.920	3.982	-4302.154	-122.099
363.15	194.053	6.167	-3874.688	-111.892
		methanol		
313.15	41.282	35.042	-1712.890	-81.171
323.15	41.801	55.051	-1124.232	-74.556
333.15	42.348	83.914	-933.461	-68.515
343.15	42.927	124.466	-787.101	-62.977
353.15	43.541	180.111	-673.360	-57.882
363.15	44.194	254.844	-583.818	-53.181
		ethanol		
313.15	59.393	19.160	-2525.630	-90.891
323.15	60.170	30.892	-1653.115	-83.181
333.15	60.993	48.237	-1369.299	-76.148
343.15	61.867	73.174	-1151.095	-69.709
353.15	62.798	108.131	-981.211	-63.792
363.15	63.794	156.030	-847.273	-58.337
		1-propanol		

313.15	76.237	7.940	-3335.793	-104.991
323.15	77.154	13.423	-2163.291	-96.212
333.15	78.115	21.836	-1785.702	-88.202
343.15	79.125	34.321	-1497.290	-80.864
353.15	80.188	52.306	-1274.200	-74.120
363.15	81.312	77.530	-1099.431	-67.901
		2-propanol		
313.15	78.207	15.599	-7008.424	-137.911
323.15	79.241	25.620	-4299.320	-127.101
333.15	80.334	40.601	-3449.121	-117.239
343.15	81.491	62.312	-2812.052	-108.208
353.15	82.720	92.917	-2329.629	-99.909
363.15	84.030	135.000	-1960.380	-92.258
		butanol		
313.15	93.304	2.510	-5022.546	-120.602
323.15	94.314	4.669	-3185.844	-110.658
333.15	95.368	8.233	-2600.572	-101.580
343.15	96.470	13.858	-2157.010	-93.262
353.15	97.623	22.384	-1816.852	-85.614
363.15	98.834	34.863	-1552.855	-78.558
		tert-butanol		
313.15	96.706	13.881	-2390.086	-104.138
323.15	98.178	23.772	-1633.440	-95.164
333.15	99.734	38.842	-1386.885	-86.957
343.15	101.382	60.910	-1196.460	-79.424
353.15	103.135	92.124	-1047.104	-72.485
363.15	105.005	134.953	-928.127	-66.075
		pentanol		
313.15	110.039	0.963	-6041.089	-135.292
323.15	111.156	1.876	-3784.796	-124.221
333.15	112.317	3.446	-3075.608	-114.113
343.15	113.525	6.017	-2542.956	-104.851
353.15	114.786	10.049	-2138.171	-96.333
363.15	116.102	16.133	-1826.825	-88.476
		2-methyl-2-pentanol		
313.15	130.516	1.876	-5367.496	-127.254
323.15	132.075	3.643	-3437.062	-116.511
333.15	133.700	6.645	-2823.552	-106.687
343.15	135.395	11.480	-2359.023	-97.671
353.15	137.167	18.916	-2002.873	-89.369
363.15	139.021	29.905	-1726.309	-81.699
		hexanol		
313.150	126.947	0.505	-6784.536	-149.981
323.150	128.157	0.973	-5363.093	-137.712
333.150	129.411	1.778	-4312.146	-126.511
343.150	130.711	3.100	-3526.763	-116.245

353.150	132.062	5.185	-2933.422	-106.804
363.150	133.467	8.358	-2480.136	-98.093
		octanol		
313.150	160.413	0.056	-10085.250	-175.697
323.150	161.918	0.126	-7848.699	-161.264
333.150	163.472	0.264	-6215.146	-148.093
343.150	165.076	0.519	-5010.545	-136.028
353.150	166.734	0.965	-4113.548	-124.936
363.150	168.450	1.708	-3438.826	-114.706
		acetaldehyde		
313.15	58.465	201.528	-1130.903	-85.003
323.15	59.588	278.503	-883.361	-78.448
333.15	60.794	376.780	-789.559	-72.417
343.15	62.097	500.027	-710.361	-66.849
353.15	63.514	652.107	-642.907	-61.694
363.15	65.064	837.036	-584.984	-56.907
		propionaldehyde		
313.15	74.643	75.929	-1517.779	-97.652
323.15	75.884	108.633	-1184.365	-90.204
333.15	77.199	151.788	-1058.588	-83.355
343.15	78.598	207.594	-952.631	-77.035
353.15	80.091	278.459	-862.550	-71.188
363.15	81.691	366.976	-785.311	-65.761
		butyraldehyde		
313.150	92.365	27.857	-2000.611	-118.239
323.150	93.700	41.725	-1770.425	-109.420
333.150	95.104	60.774	-1579.330	-101.310
343.150	96.584	86.321	-1419.057	-93.828
353.150	98.147	119.856	-1283.346	-86.905
		valeryaldehyde		
313.15	108.710	9.337	-3582.060	-127.712
323.15	109.913	14.655	-2718.043	-117.677
333.15	111.170	22.290	-2399.614	-108.479
343.15	112.486	32.961	-2135.063	-100.018
353.15	113.867	47.516	-1913.078	-92.211
363.15	115.319	66.939	-1725.077	-84.984
		capronaldehyde		
313.15	125.435	3.250	-5405.486	-142.932
323.15	126.704	5.384	-4034.592	-131.584
333.15	128.024	8.597	-3535.168	-121.192
343.15	129.400	13.285	-3123.272	-111.641
353.15	130.837	19.930	-2780.126	-102.834
363.15	132.339	29.115	-2491.545	-94.689
		2-pentanone		
313.15	313.15	313.15	313.15	313.15
323.15	323.15	323.15	323.15	323.15

333.15	333.15	333.15	333.15	333.15
343.15	343.15	343.15	343.15	343.15
353.15	353.15	353.15	353.15	353.15
363.15	363.15	363.15	363.15	363.15
		3-pentanone		
313.15	108.308	10.135	-3413.169	-131.200
323.15	109.691	15.736	-2610.054	-121.150
333.15	111.135	23.707	-2312.771	-111.921
343.15	112.646	34.757	-2065.128	-103.417
353.15	114.230	49.722	-1856.797	-95.557
363.15	115.894	69.564	-1679.921	-88.271
		cyclopentanone		
313.15	90.405	3.012	-5117.209	-146.701
323.15	91.370	4.948	-3770.346	-136.201
333.15	92.368	7.862	-3283.415	-126.568
343.15	93.401	12.123	-2884.118	-117.699
353.15	94.472	18.191	-2553.525	-109.507
363.15	95.582	26.629	-2277.318	-101.919
		cyclohexanone		
313.15	105.821	1.183	-7342.311	-165.819
323.15	106.956	1.997	-5347.099	-153.955
333.15	108.131	3.255	-4632.203	-143.075
343.15	109.347	5.143	-4049.171	-133.063
353.15	110.608	7.899	-3568.989	-123.820
363.15	111.918	11.823	-3169.821	-115.261
		1.4-dioxane		
313.15	87.045	10.482	-2041.138	-126.837
323.15	88.038	16.220	-1614.791	-117.674
333.15	89.068	24.367	-1455.554	-109.261
343.15	90.140	35.642	-1321.733	-101.509
353.15	91.257	50.892	-1207.958	-94.345
363.15	92.421	71.090	-1110.192	-87.705
		tetrahydrofurane		
313.15	83.475	41.748	-1587.067	-111.596
323.15	84.555	60.776	-1261.787	-103.507
333.15	85.686	86.236	-1138.471	-96.064
343.15	86.873	119.565	-1034.109	-89.191
353.15	88.120	162.344	-944.907	-82.827
363.15	89.435	216.289	-867.959	-76.918
		ethyl acetate		
313.15	100.492	25.209	-2115.930	-116.682
323.15	101.916	38.055	-1673.332	-107.550
333.15	103.414	55.830	-1505.330	-99.163
343.15	104.992	79.833	-1363.057	-91.435
353.15	106.661	111.538	-1241.390	-84.290
363.15	108.431	152.599	-1136.404	-77.667

		benzene		
313.15	91.001	21.917	-1576.207	-121.362
323.15	92.101	33.639	-1284.552	-112.672
333.15	93.247	49.936	-1172.732	-104.673
343.15	94.446	71.970	-1077.223	-97.287
353.15	95.700	101.026	-994.748	-90.446
363.15	97.016	138.501	-922.823	-84.092
		toluene		
313.15	108.259	7.943	-2614.234	-139.615
323.15	109.524	12.309	-2066.994	-129.464
333.15	110.839	18.511	-1862.886	-120.133
343.15	112.206	27.094	-1691.505	-111.527
353.15	113.630	38.700	-1545.918	-103.566
363.15	115.115	54.066	-1420.914	-96.181
		p-xylene		
313.15	126.693	2.635	-4103.540	-156.116
323.15	128.003	4.301	-3160.578	-144.514
333.15	129.360	6.785	-2816.003	-133.864
343.15	130.767	10.378	-2530.395	-124.056
353.15	132.228	15.436	-2290.802	-114.994
363.15	133.746	22.389	-2087.562	-106.597
		(1-methylethyl)benzene		
313.15	141.736	1.532	-5133.091	-165.932
323.15	143.187	2.561	-3910.729	-153.539
333.15	144.686	4.127	-3467.943	-142.162
343.15	146.238	6.437	-3102.970	-131.684
353.15	147.846	9.747	-2798.462	-122.002
363.15	149.514	14.370	-2541.514	-113.030
		1.3.5-trimethylbenzene		
313.15	141.600	0.839	-6241.781	-169.638
323.15	142.988	1.448	-4683.020	-156.685
333.15	144.422	2.406	-4122.995	-144.817
343.15	145.906	3.860	-3663.925	-133.905
353.15	147.442	6.002	-3283.055	-123.838
363.15	149.035	9.075	-2963.485	-114.523

		chlorobenzene		
313.15	103.699	3.687	-3820.594	-149.449
323.15	104.743	5.870	-2896.384	-138.853
333.15	105.821	9.056	-2560.812	-129.120
343.15	106.937	13.578	-2284.208	-120.150
353.15	108.093	19.839	-2053.648	-111.857
363.15	109.292	28.319	-1859.444	-104.168
		chloroform		
313.15	82.295	47.217	-1351.536	-105.990
323.15	83.452	68.693	-1089.300	-98.173

333.15	84.665	97.388	-988.890	-90.982
343.15	85.940	134.897	-903.319	-84.346
353.15	87.284	182.968	-829.649	-78.204
363.15	88.703	243.489	-765.636	-72.502
		acetonitrile		
313.15	53.771	22.601	-4723.273	-115.554
323.15	54.549	33.638	-3420.697	-106.939
333.15	55.364	48.829	-2947.038	-99.036
343.15	56.220	69.285	-2558.171	-91.762
353.15	57.122	96.297	-2236.388	-85.045
363.15	58.072	131.331	-1968.114	-78.824
		pyridine		
313.15	82.145	6.036	-2288.292	-132.422
323.15	83.082	9.599	-1785.187	-123.106
333.15	84.051	14.774	-1600.284	-114.549
343.15	85.056	22.084	-1446.412	-106.662
353.15	86.100	32.150	-1316.795	-99.371
363.15	87.184	45.700	-1206.376	-92.611
